# Nonparametric richness estimators Chao1 and ACE must not be used with amplicon sequence variant data

**DOI:** 10.1093/ismejo/wrae106

**Published:** 2024-06-13

**Authors:** Yongcui Deng, Alexander K Umbach, Josh D Neufeld

**Affiliations:** Department of Geography, Nanjing Normal University, No. 1 Wenyuan Road, Nanjing, Jiangsu Province 210023, China; Department of Biology, University of Waterloo, Waterloo, Ontario N2T 1P5, Canada; Department of Biology, University of Waterloo, Waterloo, Ontario N2T 1P5, Canada; Department of Biology, University of Waterloo, Waterloo, Ontario N2T 1P5, Canada

**Keywords:** Chao1, ACE, ASV, amplicon sequence variant, OTU, operational taxonomic units, DADA2, deblur, QIIME2, UNOISE

Alpha diversity metrics are used by microbial ecologists to quantify species richness and evenness from small subunit ribosomal RNA (SSU rRNA) gene sequence data. Although various parametric or non-parametric diversity indices may be selected for measuring sample profile diversity, the commonplace nonparametric richness estimators Chao1 and ACE must not be used for estimating total richness of amplicon sequence variant (ASV) datasets, especially when generated by algorithms that remove singletons. This perspective explains why using these richness estimators with ASV data leads to meaningless results.

The nonparametric Chao1 index [1] estimates total species richness with an equation that generates a ratio of the number of singletons squared divided by the number of doubletons multiplied by two (Box 1). This value is then added to the number of observed species to generate a species richness prediction. Within the context of microbiome datasets, each “species” is commonly represented by an ASV with a representative sequence, along with an associated read count representing abundance, and it is from these data that richness is estimated. A bias-corrected version of the Chao1 calculation is now widely preferred, and default in QIIME2 [2] and mothur [3], because it solves the issue of division by zero if no doubletons are present in the data, although this is unlikely given high depths of coverage commonly applied to samples. Similar to Chao1, the ACE richness estimator [4] depends on the total number of singleton species, but also incorporates the number of relatively abundant species (i.e. read counts >10) and rare species (i.e. read counts ≤10), along with sample coverage and the coefficient of variation for rare species (Box 1). Thus, both of these richness estimators are highly dependent on rare taxa abundances present in count data from sampled microbial communities.

Although operational taxonomic units (OTUs) were commonplace for clustering SSU rRNA gene sequences, most approaches now resolve sequences to ASVs instead. These “denoising” methods distinguish sequences by as little as one nucleotide, producing ASVs that provide a higher resolution compared to 97% OTUs. In doing so, ASVs have improved reusability across studies, sensitivity to population abundance changes, and direct comparability across datasets [5]. Increasingly, and since ~2019 when ASVs became commonplace, many articles have been published annually employing ASV data to calculate Chao1 (Fig. 1) and ACE (data not shown). However, the application of traditional richness estimators, such as Chao1 and ACE, must not be used with ASV data that have had singletons removed because this abundance category is essential for these richness estimate calculations.

**Figure 1:**
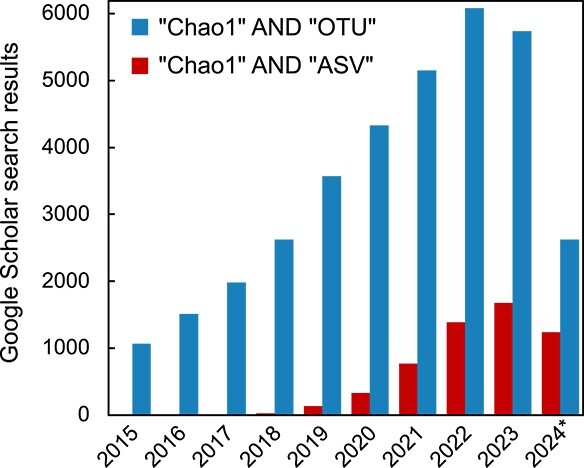
Google scholar search results for the number of publications in each year range from 2015 to 2024 (^*^ indicates that results were for search results available June 10, 2024), retrieved using Boolean searches: [“Chao1” AND “OTU”] or [“Chao1” AND “ASV”].

Commonly used denoising algorithms, such as DADA2 [6] and Deblur [7], are used for processing amplicon sequence reads and generating ASVs. The DADA2 algorithm calculates sequence error rates using run-specific sequence quality scores, while Deblur relies on a set of precalculated values for determining misread probability. Both algorithms combine this information with read frequency and abundance data to determine whether a given read is legitimate or an artifact. However, these algorithms are impeded by technical limitations intrinsic to Illumina amplicon data that prevent confident resolution of authentic singleton sequences. As such, removal of all ASVs supported by a single sequence is the default setting. For example, when using the default DADA2 pipeline, singletons are deleted when denoising the forward and reverse pairs of sequences, respectively. Although singleton ASVs will occasionally be generated following read merging in paired-end datasets, these singletons represent pairings of forward and reverse reads that were unable to be resolved in isolation, are just as likely to be erroneous, and are thus recommended for removal.

Box 1 1. Two formulas for Chao1

${S}_{chao1}={S}_{obs}+\frac{F_1^2}{2{F}_2}$

${S}_{chao1}={S}_{obs}+\frac{F_1\left({F}_1-1\right)}{2\left({F}_2+1\right)}$

*S_obs_* represents the total number of observed species;

*F*_1_ refers to the number of singleton species;

*F*_2_ refers to the number of doubleton species.

2. Formula for ACE

${S}_{ACE}={S}_{abund}+\frac{S_{rare}}{C_{ACE}}+\frac{F_1}{C_{ACE}}{\gamma}_{ACE}^2$

${\gamma}_{ACE}^2=\mathit{\max}\left[\frac{S_{rare}{\sum}_{i=1}^{10}i\left(i-1\right){F}_i}{C_{ACE}{N}_{rare}\left({N}_{rare}-1\right)}-1,0\right]$

${C}_{ACE}=1-\frac{F_1}{N_{rare}}$

${N}_{rare}={\sum}_{i=1}^{10}i{F}_i$

*S_abund_* represents the total number of abundant species (abundance > threshold, e.g. 10);

*S_rare_* represents the total number of rare species (1 ≤ abundance ≤ threshold of 10);

*F*_1_ refers to the number of singleton species;

*C_ACE_* is the estimated value of sample coverage;

γ^2^*_ACE_* is the coefficient of variation for rare species.

Some singleton sequences can be retained by pooling samples during ASV generation by the DADA2 algorithm. This option shares read information among samples during ASV inference and will retain singletons that appear in more than one sample. Therefore, the pooling sample pipeline will result in higher richness estimates compared to non-pooled samples [8]. This option is computationally more demanding, detects only few additional sequences, and is not default within DADA2 or QIIME2, and thus rarely used. Regardless, using pooled-sample ASV generation does not prevent singletons from being removed if they are detected in a single sample, thus the fundamental issue remains.

The inability of denoising algorithms to confidently resolve true singleton ASVs, in addition to default and/or mandatory removal of singletons (i.e. default removal in the R version of DADA2; default and mandatory removal in the QIIME2 version of DADA2), renders the use of singleton-dependent Chao1 and ACE metrics meaningless and thus unacceptable for alpha diversity analyses for ASV-generated datasets. In addition, ASV datasets are often standardized for sequencing depth among samples by rarefying or using an equivalent method (e.g. ranked subsampling). Although the decision to rarefy ASV datasets is itself an area of active debate [9, 10], the result of rarefying is the generation of new count data that will contain singletons arising from the process of subsampling itself, which will be influenced by the original sequencing depths of samples, and does not resolve the underlying problem associated with ASV generation. Ultimately, any data processing resulting in the removal of rare ASVs will bias alpha diversity estimates [11]. Thus, ASV generation pipelines that automatically delete rare taxa necessary for Chao1 and ACE richness estimators will result in nonsensical estimates that are not ecologically or mathematically relevant and should not be presented or interpreted.

As an alternative to mandatory removal of singletons, DADA2 in the R version [6], Deblur [7], and UNOISE [12] provide options to set minimum ASV abundance thresholds, with defaults of 2, 10, and 8, respectively, that allow users to retain singletons and other relatively rare ASV sequences. However, for high-throughput sequence data, and clone library sequences prior to “next-generation” sequencing, rare taxa will be increasingly associated with sequencing errors, PCR bias, chimeras, and other technical artifacts. Because a particularly large proportion of singleton sequences represent artifacts [13], it would be unwise to perform diversity calculations based on the inclusion of singleton ASV data. However, for many diversity metrics, this bias will be common to all samples, leading to global overestimation from true values, yet still permitting relative comparisons of diversity among samples and treatments. This could also be argued for Chao1 and ACE richness estimators, but any use of these metrics with ASV data would require specifying in the accompanying methods section that rare ASVs were retained and justify the value of using these estimates given such high sensitivity to the prevalence of artifacts in the resulting data.

Because Chao1 and ACE calculations must not be performed on singleton-removed ASV data, and their use with singleton-retained ASV data is highly questionable, an alternative approach would be to generate OTUs, using pipelines such as mothur [3] or UPARSE [14], for the specific purpose of analyzing alpha diversity. This would be appropriate given that sequencing artifacts will be less impactful on the resulting data (e.g. for 97% OTUs), low abundance taxa are retained, and the biological units captured by 97% OTUs may offer resolution akin to genus (or species) levels often desired by microbial ecologists. In addition, when calculating these alpha diversity metrics, rarefaction has been recommended to help address uneven sequencing effort [10, 15]. For those continuing to use more typical and commonplace ASV generation with default pipeline settings, other diversity metrics that rely less on specific counts of singletons or doubletons (e.g. observed ASVs, Faith’s phylogenetic diversity, Shannon index, and Simpson index) should be used instead of generating Chao1 and ACE estimates.

## Data Availability

Data sharing not applicable to this article as no datasets were generated for the current study.
